# Prevalence of nephropathy among patients with diabetes mellitus in Africa: a systematic review and meta-analysis

**DOI:** 10.3389/fcdhc.2025.1551088

**Published:** 2025-04-25

**Authors:** Grace I. Adebayo-Gege, Peter Ifeoluwa Adegbola, Lawrence Dayo Adedayo, Adegboyega Moses Oyefabi, Ifeoluwa Temitayo Oyeyemi, Odeniran Olubukola, Adewale Adegboyega Oke, Oluchukwu Perpetual Okeke, Olunike Rebecca Abodunrin, Folahanmi Tomiwa Akinsolu, Olajide Odunayo Sobande

**Affiliations:** ^1^ Department of Physiology, Faculty of Basic Medical Sciences Baze University, Abuja, Nigeria; ^2^ Department of Biochemistry and Forensic Science, Faculty of Natural and Applied Sciences, Abiola Ajimobi Technical University, Ibadan, Nigeria; ^3^ Department of Human Physiology, Faculty of Basic Medical Sciences, College of Health Sciences, Federal University Wukari, Wukari, Nigeria; ^4^ Department of Community Medicine, Kaduna State University, Kaduna, Nigeria; ^5^ Department of Biosciences and Biotechnology, University of Medical Sciences, Ondo, Nigeria; ^6^ Department of Medicinal Chemistry & Quality Control, National Institute for Pharmaceutical Research and Development (NIPRD), Abuja, Nigeria; ^7^ Department of Medical Laboratory Science, McPherson University, Seriki Sotayo, Ogun State, Nigeria; ^8^ Nigerian Institute of Medical Research Foundation, Yaba, Lagos State, Nigeria; ^9^ Clinical Sciences Department, Lead City University, Ibadan, Nigeria

**Keywords:** nephropathy, Africa, diabetes mellitus, hypertension, prevalence

## Abstract

**Background:**

Diabetic nephropathy (DN) is one of the most frequent microvascular consequences of diabetes, accounting for a significant portion of morbidity and mortality in diabetic patients in Africa. This study aims to report on the prevalence of nephropathy among patients with diabetes mellitus patients in Africa and the risk factors.

**Methods:**

This systematic review was reported using Preferred Reporting Items for Systematic Reviews and Meta-Analyses (PRISMA) standards, and the protocol was pre-registered in PROSPERO with the registration number CRD42024587467. The search was conducted across databases such as PubMed, Google Scholar, CINAHL and Scopus to retrieve studies published between January 2000 and August 2024. All statistical analyses were conducted using R software (version 4.4.2). The pooled prevalence of nephropathy in patients with diabetes was calculated with a 95% confidence interval (CI).

**Results:**

Thirty-four (34) articles met the inclusion criteria. Only 28 studies were incorporated into the meta-analysis to determine the pooled prevalence of nephropathy among diabetes patients. The findings indicated a pooled prevalence of 21% (95%, CI: 16-28) of nephropathy among diabetes patients. Among type 1 and type 2 diabetes patients, the pooled prevalence of nephropathy is 46% (95%, CI: 18-77, I² = 98%) and 20% (95% CI: 14-27, I² = 98%), respectively. Weighted prevalence of 47%, 31%, 33% and 11% were reported in North Africa, Central Africa, South Africa and West Africa respectively. The result also showed that diabetes patients with hypertension are more than three times at risk of developing nephropathy compared to those without hypertension OR:3.46 (95% CI: 2.61-4.59).

**Conclusion:**

The current study showed the prevalence of nephropathy with a significant association with hypertension among diabetic mellitus patients. Higher prevalence in North Africa is likely due to Western cultural impacts on dietary consumption.

**Systematic Review Registration:**

https://www.crd.york.ac.uk/prospero/, identifier CRD42024587467.

## 1 Introduction

Diabetes mellitus (DM) is a chronic metabolic disorder characterized by either insulin deficiency due to the destruction of pancreatic islet beta cells or by insulin resistance, where the body fails to use the insulin available (1) effectively. With the global prevalence of DM reaching 10.5% among adults aged 20 to 79 impacting approximately 440 million people—diabetes has become a critical public health issue worldwide (2). Projections suggest that over 550 million people will be affected by 2035, intensifying the urgency for effective management and prevention of its complications (2, 3).

Diabetic nephropathy (DN), a microvascular complication of diabetes, significantly contributes to morbidity and mortality among individuals with diabetes (4–6). DN is a leading cause of end-stage renal disease (ESRD) globally, accounting for 12% to 55% of all ESRD cases (4, 7, 8). While it affects 30-40% of those with type 1diabetes and 10-20% of those with type 2 diabetes (9), the sheer prevalence of type 2 diabetes means that the majority of ESRD cases occur in this population. Additionally, age-related decline in kidney function and comorbid conditions, such as hypertension, further elevate the risk of kidney complications in type 2 diabetes patients, particularly in older adults (10).

While several studies have assessed chronic kidney disease (CKD) prevalence in African populations, results have varied across countries and patient groups, highlighting the complex interaction between diabetes and nephropathy in the region. Studies in Ethiopia (11–14), Nigeria (15, 16), Ghana (10, 17), Tanzania (18), and the Democratic Republic of Congo (19) report a range of CKD prevalence among people with diabetes, yet these data are often fragmented and inconsistent in methodology. Furthermore, risk factors, demographic influences, and regional variations within Africa remain underexplored comprehensively and systematically.

This systematic review and meta-analysis aim to fill this gap by determining the prevalence of nephropathy among diabetic patients across African nations. By synthesizing available evidence, we examined the region-specific risk factors that potentiate DN incidence among patients with DM in Africa, offering critical insights for regional healthcare planning and targeted interventions.

## 2 Methods

### 2.1 Protocol registration

This systematic review was reported using Preferred Reporting Items for Systematic Reviews and Meta-Analyses (PRISMA) standards. The protocol was pre-registered in PROSPERO with the registration number CRD42024587467.

### 2.2 Review question

What is the prevalence of nephropathy among patients with diabetes mellitus in Africa?

What are the factors contributing to the prevalence of nephropathy among patients with diabetes mellitus in Africa?

### 2.3 Main study outcomes

#### 2.3.1 Primary outcome

1. Pooled prevalence estimates of diabetes nephropathy among diabetes mellitus patients
2. Risk factors associated with nephropathy in Africa

#### 2.3.2 Secondary outcomes

1. Regional prevalence of nephropathy among diabetes patients
2. Prevalence of diabetes nephropathy by diabetes mellitus type
3. Prevalence of diabetes nephropathy based on gender of diabetes patients

### 2.4 Search strategy

The PICO framework was utilized before searching for relevant articles to frame and structure the research concepts and develop the search terms. Each component of the PICO framework and the search terms are shown in **Table 1**. The search strategy involves utilizing specific keywords across various databases, such as PubMed, Google Scholar, CINAHL (Cumulative Index to Nursing and Allied Health Literature), and Scopus, to retrieve relevant literature.

**Table 1:** Search terms using the PIO framework.

PIO	
Patient	All studies reporting nephropathy among diabetes mellitus patients
Intervention	Studies reporting Albuminuria: Microalbuminuria (30-300 mg/g), macroalbuminuria (>300 mg/g), and Reduced eGFR: <60 among diabetes patients
Outcome	Prevalence of diabetes nephropathy among diabetes mellitus patients Risk factors associated with nephropathy in Africa Regional prevalence of nephropathy among diabetes patients Prevalence of diabetes nephropathy by diabetes mellitus type

The search across the databases involved careful formulation of search queries combined with Boolean operators like ‘AND’’ and ‘OR.’’ The search queries were thereafter submitted into the selected databases to retrieve studies published between January 1, 2000, and August 31^st^, 2024, on the prevalence of nephropathy among diabetes patients in Africa. The search included all studies published in African lingua franca. The search strategy is shown in **Supplementary Table 1**.

### 2.5 Inclusion and exclusion criteria

The systematic review included Cross-sectional studies, Cohort studies (prospective or retrospective), and Case-control studies. Studies that met the eligibility criteria were included and uploaded to Rayyan, a collaborative systematic literature review web tool (20). Before the article screening, the level of agreement between the reviewers was evaluated using kappa scores. Thereafter, two independent reviewers (G.I.G., P.I.A) removed duplicate articles and screened the titles and abstracts for suitability before the full-text screening. In contrast, the third reviewer (L.D.A) resolved conflicts through discussion or consultation. A similar approach was used for full-text screening. The list of excluded studies is provided in **Supplementary Table 2**.

### 2.6 Study selection and screening

The selection of studies was based on preset inclusion and exclusion criteria. In addition to the study designs already described will be included, studies conducted in hospital settings, community-based, studies with nephropathy directly related to diabetes as an outcome, population ≥100, studies that clearly define nephropathy, provide quantitative data on the prevalence of diabetes nephropathy, reports on the contributing/risk factors for nephropathy and prevalence, conducted among Africans were selected. Articles published in other languages aside from English were translated into English with the help of a translator, while articles not freely available were retrieved using a university subscription. Overall, the article selection followed a strict process to answer the research questions and achieve the objectives.

### 2.7 Data extraction

Data extraction was performed using a pre-tested form prepared in Microsoft Excel. The reviewers (G.I.G., P.I.A, L.D.A) extracted information including article title, first author’s surname, publication year, country, geographical region of the study, study setting, sample size, sample size by gender, study design, the mean age of participants, type of diabetes, duration of diabetes, nephropathy definition, number of confirmed nephropathy cases, risk factors, number of controlled and uncontrolled diabetes from the selected articles in the **Supplementary Table 3**.

### 2.8 Assessment of risk of bias in included studies

The methodology quality of the included studies was assessed by adapting the JBI critical appraisal checklist for studies reporting prevalence data (21). Two reviewers assessed methodological quality and risk of bias in the included studies, while a third reviewer resolved discrepancies. The nine-quality domain of the assessment checklist was ranked to score the included articles as either high, moderate, or low in **Supplementary Table 4.**

### 2.9 Dealing with missing data

For missing data in included studies, such as failure to report a particular outcome, such study was excluded from the analysis of such outcome. When a study has missing data and such data are unavailable, we conduct subgroup analyses to explore how missing data might influence results.

### 2.10 Statistical analysis

All statistical analyses were conducted using R software (version 4.4.2). The pooled prevalence of nephropathy in patients with diabetes was calculated with a 95% confidence interval (CI). When at least two studies from the same region reported nephropathy prevalence among diabetes patients, a weighted prevalence was used to determine the overall prevalence in each region. Odds ratios (OR) were utilized to explore potential risk factors, including underlying hypertension and a duration of diabetes greater than 10 years.

Statistical heterogeneity among studies was assessed using Cochran’s Q test and the I² statistic. A P-value of <0.1 for the Q test and an I² value greater than 50% indicated statistically significant heterogeneity. Given the anticipated heterogeneity, a random-effects model was employed to pool all outcomes, providing a more conservative estimate of prevalence. Kappa statistics were used to assess inter-rater agreement between reviewers during the study inclusion process.

### 2.11 Publication bias and sensitivity analysis

Funnel plots were used to assess publication bias for the primary outcome. A leave-one-out analysis, systematically excluding each study, was performed to evaluate the robustness of the results and assess the impact of individual studies on the pooled prevalence.

## 3 Results

### 3.1 Selection of studies

**Figure 1:**
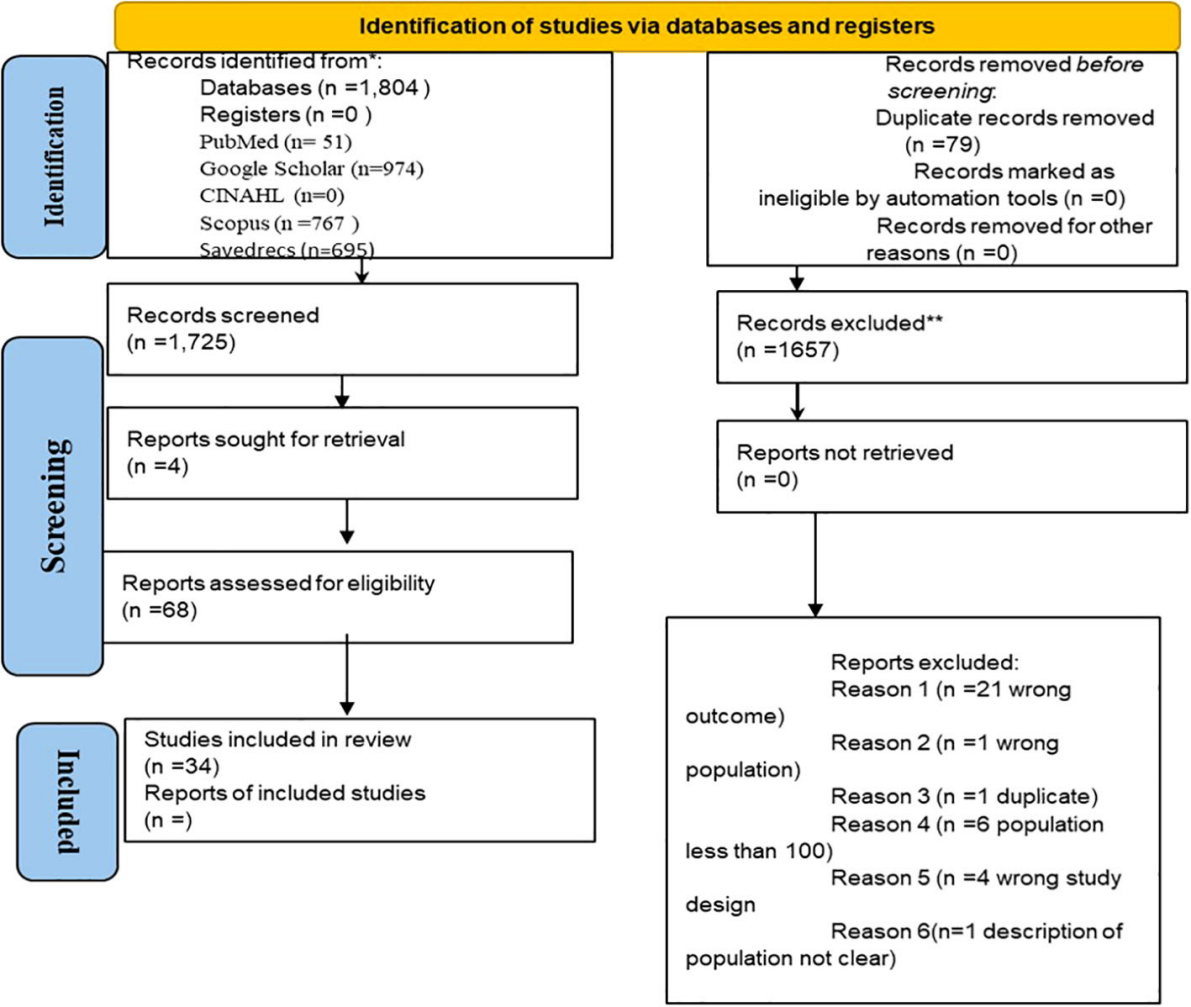
PRISMA flow chart for the search results.

Electronic searches retrieved one thousand eight hundred and four (1,804) records (see **Figure 1**). Seventy-nine (79) duplicate records were removed, leaving 1,725 articles for screening. A total of 1,657 articles were excluded based on the title and abstract, while the full-text record of the remaining 68 studies was obtained for full-text evaluation. Based on population size, ambiguity of study design and the study outcome, 34 were excluded. The selected 34 articles met the inclusion criteria, clearly revealing the study population and prevalence of diabetic nephropathy (See **Figure 1**).

### 3.2 Characteristics of the articles included in this study

The characteristics of the articles included in this study were summarized in **Tables 2**, **3**. The thirty-four (34) articles were published in 2002 and 2024. Of all the studies, 25 (73.5%) were cross-sectional designs (22–30, 32–40, 42, 44, 47, 49, 52, 53, 55), 5 (14.7%) were retrospective follow up (45, 46, 50, 51, 54), 2 (5.9%) were cohort (31, 48) and 2 (5.9%) case-control studies (41, 43) (See **Table 3**). Most of the included studies were facility-based studies.

**Table 2:** Summary of studies included.

S/N	First Author’s Surname	Year	Country	Geographical region	Study settings	Sample size	Male	Female	Mean age	Type of study	Type of Diabetes	Nephropathy definition	Cases of nephropathy
1.	Adeniyi and Owolabi (22)	2020	South Africa	South Africa	Facility based	327	97	230	≥30	Cross- sectional study	type 2	Albuminuria, reduced eGFR	80
2.	Alami et al (23)	2022	Morocco	North Africa	Facility based	505	75	430	57.27 ± 10.74	Cross-sectional study	type 2	Albuminuria, reduced eGFR	50
3.	Ahmed et al (24)	2017	Sudan	Northeast Africa	Facility based	316	185	131	58 ± 10	Descriptive Cross-sectional	Not specified	Albuminuria, reduced eGFR	94
4.	Ahmed et al (25)	2020	Sudan	North East Africa	Facility based	100	39	61	>10	Cross sectional study	type 1	Albuminuria, reduced eGFR	36
5.	Ritah Kiconco et al (26)	2019	Uganda	East Africa	Facility based	140	45	95	45-54	Cross sectional study	Type 2	Albuminuria, reduced eGFR	32
6.	Adem et al (27)	2024	Ethiopia	East Africa	Facility based	267	163	104	≥ 18	Cross-sectional study	type 1	Albuminuria, reduced eGFR	236
7.	Wanjohi et al (28)	2002	Kenya	East African	Facility based	100	31	69	53.7 ± 9.3	Cross sectional study	type 2	Albuminuria, reduced eGFR	26
8.	Muddu et al (29)	2019	Uganda	East African	Facility based	175	90	85	46 ± 15	Cross sectional study	Type 2	Albuminuria	64
9.	Choukem et al (30)	2012	Cameroon	Central Africa	Facility based	420	207	213		Cross-sectional study	Type 2	Albuminuria	130
10.	Bentata et al (31)	2015	Morocco	North Africa	Facility based	637	240	397	58.5 ± 10.8	Prospective Cohort study	Type 2	Albuminuria, Kidney biopsy, reduced eGFR	492
11.	Mhundwa et al (32)	2023	South Africa	South Africa	Facility based	224	81	163	62.5	Cross-sectional study	Type 2	reduced eGFR	61
12.	Damtie et al (33)	2018	Ethiopia	East Africa	Facility based	229	114	115	47 ± 15.7	Cross-sectional study	Type 2 and Type 1	Albuminuria, Kidney biopsy, reduced eGFR	55
13.	Aboelnasr et al (34)	2020	Egypt	North Africa	Facility based	153	114	115	49.1	Cross-sectional study	Type 2 and Type 1	Albuminuria, Kidney biopsy, reduced eGFR	[70
14.	Otieno et al (35)	2020	Kenya	East Africa	Facility based	385	133	252	63.3	Cross-sectional study	Type 2	Albuminuria, Kidney biopsy, reduced eGFR	385
15.	Taderegew (55)	2020	Ethiopia	East Africa	Facility based	422	193	229		Cross-sectional study		reduced eGFR	192
16.	Molefe-Baikai et al (36)	2020	Botswana	East Africa	Facility based	289	98	191	42–53	Cross-sectional study	Type 2	Albuminuria, Kidney biopsy, reduced eGFR	129
17.	Ephraim et al (37)	2016	Ghana	West Africa	Facility based	200	39	161		Cross-sectional study		Albuminuria, Kidney biopsy, reduced eGFR	74
18.	Worku et al (38)	2010	Ethiopia	East Africa	Facility based	305	192	113		Cross-sectional study	TYPE 2 and Type 1	Albuminuria	48
19.	Abdulkadr et al (39)	2022	Ethiopia	East Africa	Facility based	362			55.4 ± 13.63	Cross-sectional study	Type 1 and 2	Kidney biopsy, reduced eGFR	53
20.	Eghan et al (40)	2007	Ghana	West Africa	Facility based	109			54.1 ± 10.9	Cross-sectional study		Albuminuria	47
21.	Adebamowo et al (41)	2016	Ghana,Kenya and Nigeria	West and East Africa	Facility and Community based	4815	1974	2841	48	Case-control	Type 2	Albuminuria, Kidney biopsy, reduced eGFR	420
22.	Israel et al (42)	2024	Ethiopia	East Africa	Facility based	626	327	299		Cross-sectional study		Kidney biopsy, reduced eGFR	17
23.	Zemicheal et al (43)	2020	Ethiopia	East Africa	Facility based	840	453	387		Case-control	type 1 and 2	NS	168
24.	Tannor et al (44)	2019	Ghana	West Africa	Facility based	388	NC	NC		Cross-sectional study	type 2	reduced eGFR	56
25.	Alebiosu et al (16)	2003	Nigeria	West Africa	facility based	465				Retrospective study	type 2	reduced eGFR	191
26.	Kebede et al (45)	2021	Ethiopia	East Africa	facility based	467	185	277		Retrospective follow-up study	type 2	reduced eGFR	63
27.	Merid et al (46)	2024	Ethiopia	East Africa	Facility based	532	292	240		Retrospective follow up study	type 2	Albuminuria, reduced eGFR	17
28.	Tesfe et al (47)	2022	Ethiopia	East Africa	Facility based	329	199	130		Cross sectional study	type 1 and 2	Age, hypertension, reduced eGFR	55
29.	Sarfo et al (48)	2019	Ghana	West Africa	Facility based	422	114	308	49.7 ± 12.2	Prospective cohort study	type 2	reduced eGFR	21
30.	Alemu et al (49)	2020	Ethiopia	East Africa	Facility based	272	137	135	51.67 ± 13.75	Cross sectional study	type 2, type 2	reduced eGFR, systosolic blood pressure	39
31.	Ahmed et al (50)	2022	Ethiopia	East Africa	Facility based	415	199	216	56.13 ± 10.2	Retrospective follow-up study	Type 2	reduced eGFR, development of cardiovascular disease	45
32.	Tamru et al (51)	2020	Ethiopia	East Africa	Facility based	346	178	168	56.70 ± 10.48	Retrospective follow-up study	type 2	reduced eGFR	68
33.	Machingura et al (52)	2017	Zimbabwe	Southern Africa	Facility based	344			57.6 ± 14.8	Cross sectional study	type 2	Albuminuria	154
34.	Hamat et al (53)	2016	Chad	Central Africa	Facility based	181	114	67	58.7	Cross-sectional study	type 2	reduced eGFR	54

**Table 3:** Summary of nephropathy risk factors in diabetes patients.

S/N	First Author’s Surname	Year	Country	Risk factors	Number of uncontrolled diabetes	number of controlled diabetes
1.	Adeniyi and Owolabi (22)	2020	South Africa	Gender, income, hypertension, smoking, Sedentary lifestyle	NS	NS
2.	Alami et al (23)	2022	Morocco	NS	342	163
3.	Ahmed et al (24)	2017	Sudan	NS	232	84
4.	Ahmed et al (25)	2020	Sudan	hypertension	90	10
5.	Ritah Kiconco et al (26)	2019	Uganda	Age, duration of diabetes	NS	NS
6.	Adem et al (27)	2024	Ethiopia	Smoking, alcohol intake	NS	NS
7.	Wanjohi et al (28)	2002	Kenya	NS	NS	NS
8.	Muddu et al (29)	2019	Uganda	NS	NS	NS
9.	Choukem et al (30)	2012	Cameroon	NS	NS	NS
10.	Bentata et al (31)	2015	Morocco	NS	442	195
11.	Mhundwa et al (32)	2023	South Africa	Gender	203	41
12.	Damtie et al (33)	2018	Ethiopia	family history of disease	127	102
13.	Aboelnasr et al (34)	2020	Egypt	family history of disease, systosolic blood pressure, hypertension, smoking	NS	NS
14.	Otieno et al (35)	2020	Kenya	Age, hypertension, systosolic blood pressure	233	152
15.	Taderegew (55)	2020	Ethiopia	Age	281	141
16.	Molefe-Baikai et al (36)	2020	Botswana	Age	204	85
17.	Ephraim et al (37)	2016	Ghana	Age, duration of diabetes	NS	NS
18.	Worku et al (38)	2010	Ethiopia	Age	NS	NS
19.	Abdulkadr et al (39)	2022	Ethiopia	Age	NS	NS
20.	Eghan et al (40)	2007	Ghana	Age, lifestyle	NS	NS
21.	Adebamowo et al (41)	2016	Ghana, Kenya and Nigeria	diabetes type	NS	NS
22.	Israel et al (42)	2024	Ethiopia	age, religion, sex, ethnicity, occupation residence, monthly income, educational and marital status	NS	NS
23.	Zemicheal et al (43)	2020	Ethiopia	age, BMI, hypertension, non-adherence to medication	373	467
24.	Tannor et al (44)	2019	Ghana	age, gender, hypertension	NS	NS
25.	Alebiosu et al (16)	2003	Nigeria	gender, hypertension	NS	NS
26.	Kebede et al (45)	2021	Ethiopia	Gender, duration of diabetes, systosolic blood pressure, anaemia, coronary heart diseases	NS	NS
27.	Merid et al (46)	2024	Ethiopia	age, gender, systolic blood pressure, and duration of diabetes	NS	NS
28.	Tesfe et al (47)	2022	Ethiopia		204	125
29.	Sarfo et al (48)	2019	Ghana		NS	NS
30.	Alemu et al (49)	2020	Ethiopia		NS	NS
31.	Ahmed et al (50)	2022	Ethiopia		NS	NS
32.	Tamru et al (51)	2020	Ethiopia	Gender	147	199
33.	Machingura et al (52)	2017	Zimbabwe		NS	NS
34.	Hamat et al (53)	2016	Chad		NS	NS

NS, Not specified.

The studies covered 12 different countries from the various geographical regions of Africa. More than half of the studies 18 (52.9%) were reported from the East Africa region. The captured East African countries included Ethiopia 13 (38.2%) (27, 33, 38, 39, 42, 43, 45–47, 49–51, 55), Uganda 2 (5.9%) (26, 29), Kenya 2 (5.9%) (28, 35) and Botswana 1 (2.9%) (36). West Africa and North Africa regions respectively had 5 (14.7%) of the studies performed in countries like Ghana 4 (11.8%) (37, 40, 44, 48) and Nigeria 1 (2.9%) (54) and North African countries like Morocco 2 (5.9%) (23, 31), Sudan 2 (5.9%) (24, 25) and Egypt 1 (2.9%) (34). Other regions covered in the selected articles are Southern Africa 3 (8.8%) with the record from South Africa 2 (5.9%) (22, 32), and Zimbabwe 1 (2.9%) (54), as well as Central Africa 2 (5.9%) region which had 1 (2.9%) (30) of the record from Cameroon and 1 (2.9%) (53) from Chad. A study conducted in sub-Saharan Africa (2.9% of total data) was drawn from three countries: Nigeria, Ghana, and Kenya.”

### 3.3 Prevalence of nephropathy among diabetes patients

**Figure 2:**
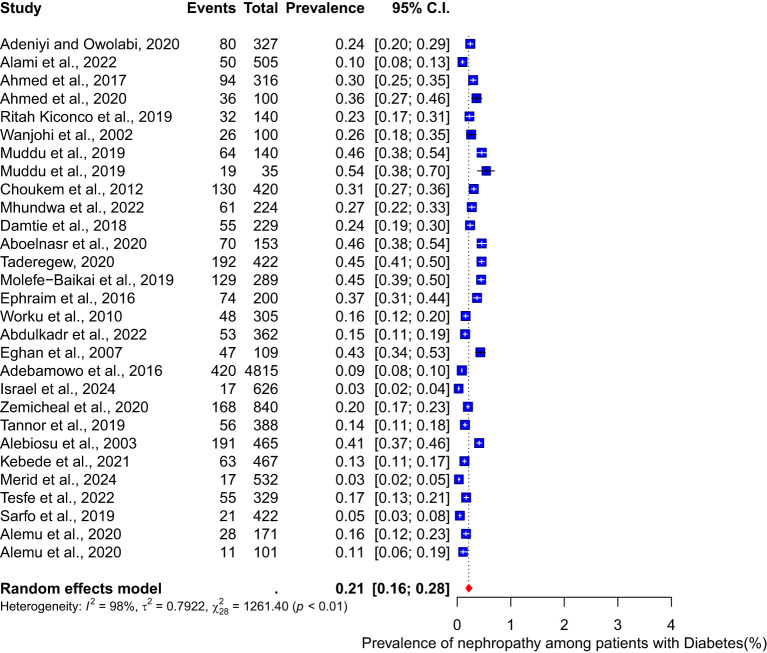
Pooled prevalence of nephropathy among diabetes patients.

This meta-analysis incorporated 28 studies to determine the pooled prevalence of nephropathy among diabetes patients. The findings indicated a pooled prevalence of 21% (95% CI 16-28) of nephropathy among diabetes patients with a substantial heterogeneity (I^2^ = 98%) (**Figure 2**).

### 3.4 Prevalence of nephropathy based on type of diabetes

**Figure 3:**
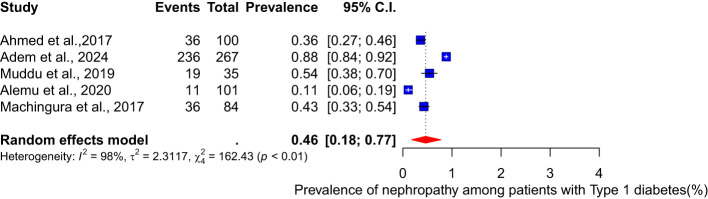
Pooled prevalence of nephropathy among Type 1 diabetes patients.

**Figure 4:**
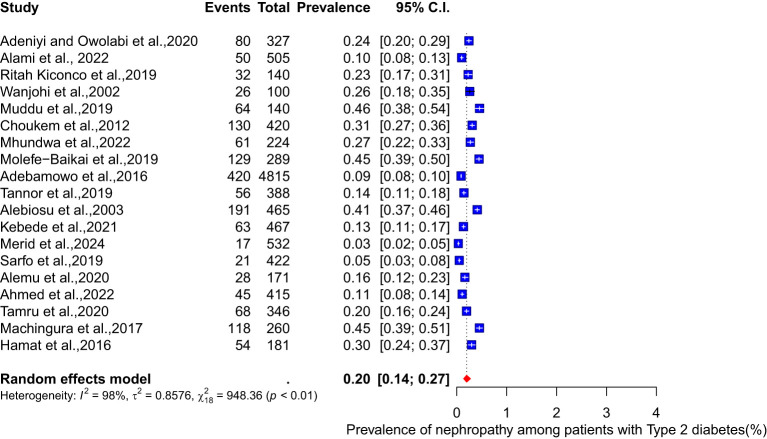
Pooled prevalence of nephropathy among Type 2 diabetes patients.

The pooled prevalence of nephropathy among patients with different types of diabetes (type 1 and type 2) was conducted. Among type 1 diabetes patients, the pooled prevalence of nephropathy is 46% (95% CI: 18-77, I² = 98%) (see **Figure 3**). For type 2 diabetes patients, the meta-analysis of eighteen studies shows a pooled prevalence of 20% (95% CI: 14-27, I² = 98%) (**Figure 4**).

### 3.5 Prevalence of nephropathy by gender

**Figure 5:**
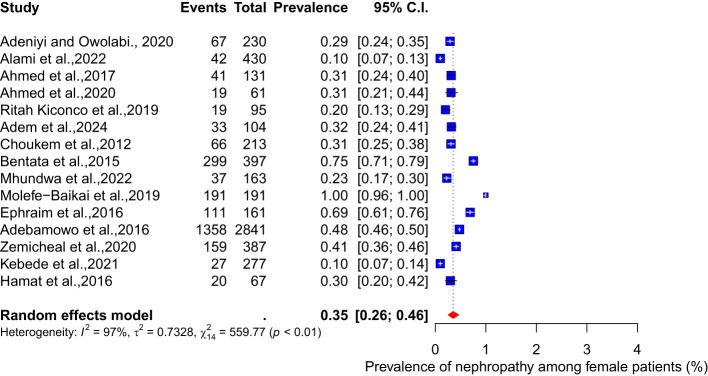
Prevalence of nephropathy among female diabetes patients.

**Figure 6:**
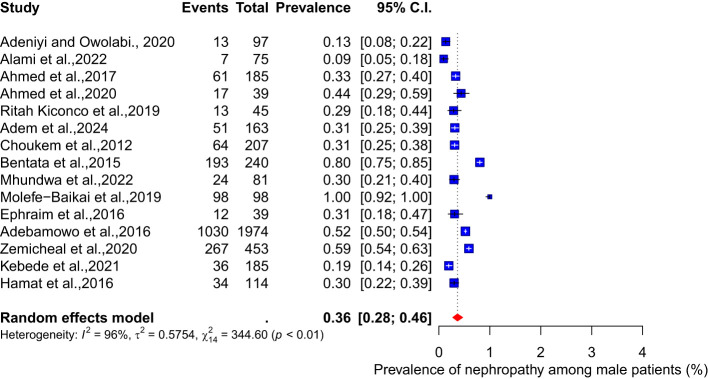
Prevalence of nephropathy among male diabetes patients.

There were no significant differences in the pooled prevalence of nephropathy between male and female diabetes patients. The meta-analysis results show a pooled prevalence of 35% (95% CI: 26-45, I² = 97%) among female diabetes patients, while the pooled prevalence among male diabetes patients is 36% (95% CI: 28-46, I² = 96%) (see **Figures 5**, **6**).

### 3.6 Prevalence of nephropathy by region

**Figure 7:**
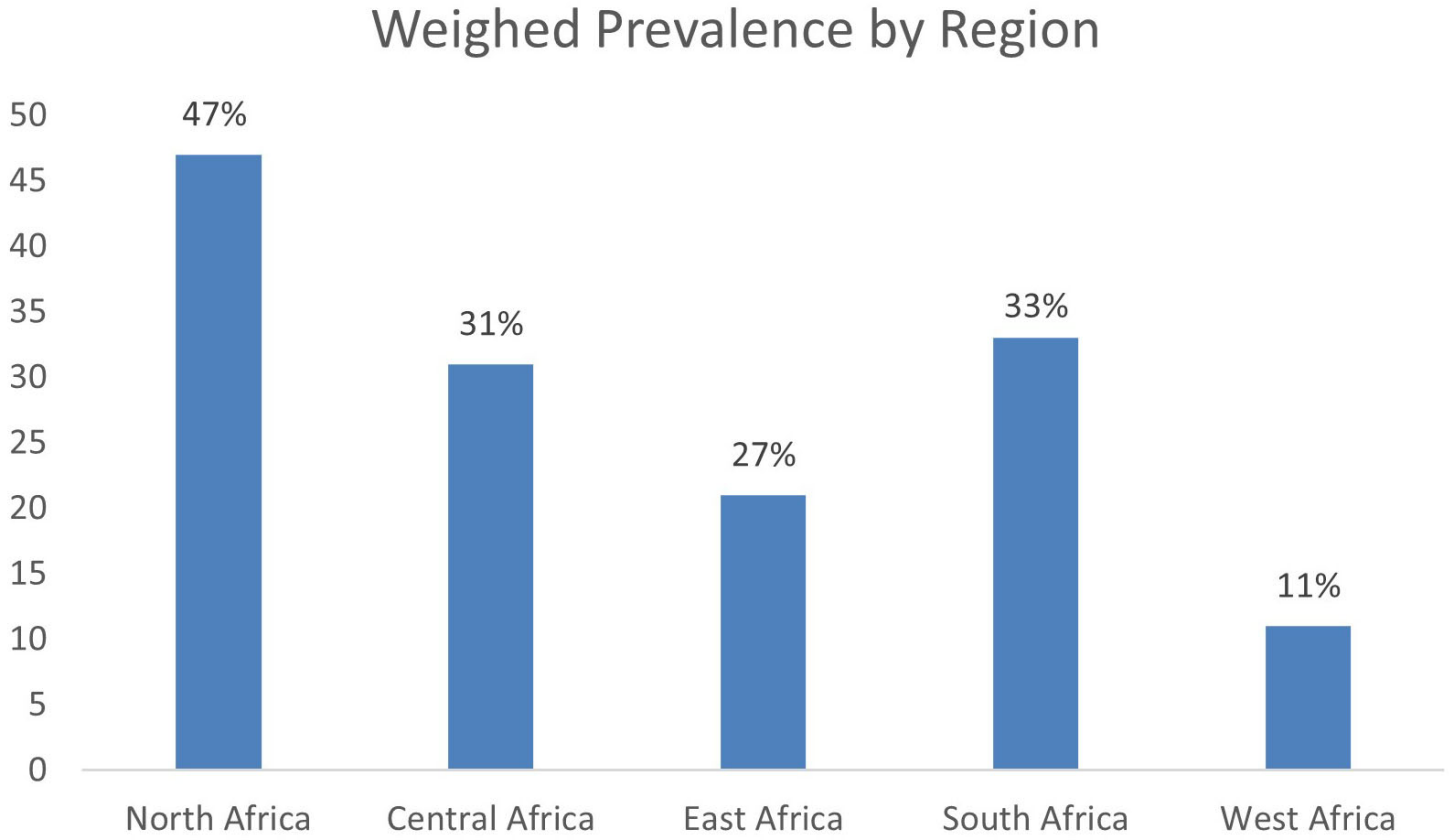
Weighted prevalence by region, all data are in percentages (%).

**Figure 8:**
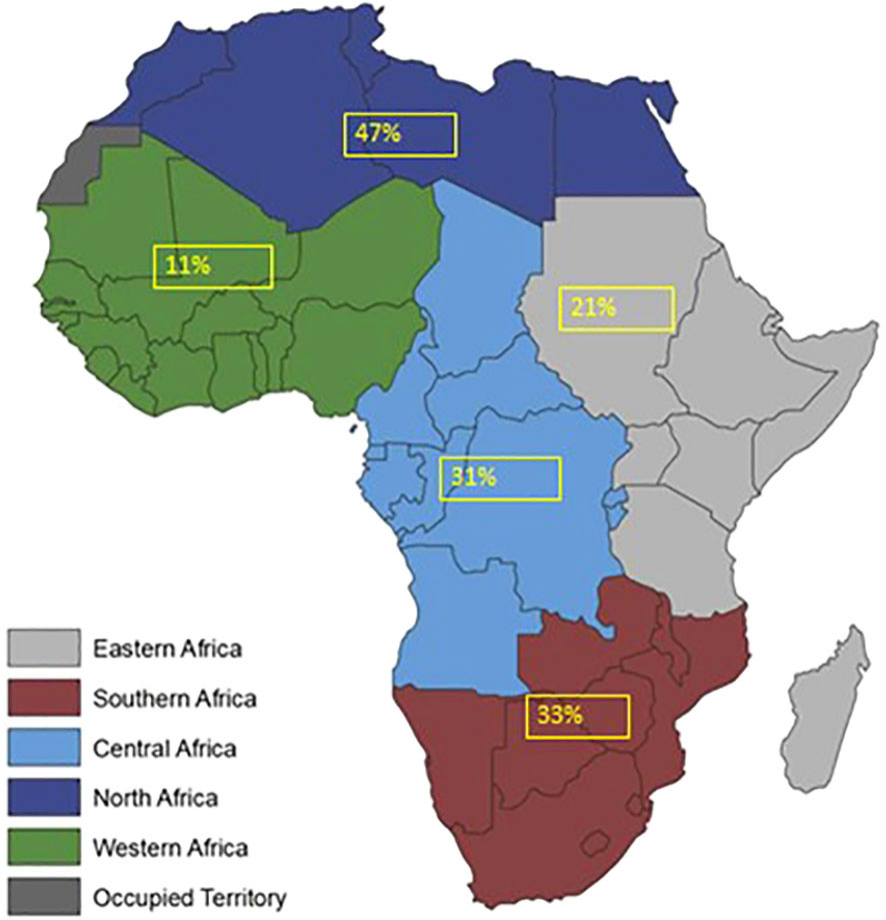
Prevalence of Nephropathy among diabetes patients by different region.

The weighted prevalence of nephropathy among diabetes patients varies across African regions. In North Africa, three studies with a total sample size of 1,295 reported a weighted prevalence of 47%. In Central Africa, two studies involving 601 participants indicated a prevalence of 31%. Eighteen studies conducted in East Africa, with a combined sample size of 5,453, found a prevalence of 21%. In South Africa, the weighted prevalence reported by four studies was 33%. Lastly, four studies in West Africa, with a total sample size of 6,090, showed a prevalence of 11% (see **Figures 7**, **8**).

### 3.7 Association between hypertension and nephropathy among diabetes patients

**Figure 9:**
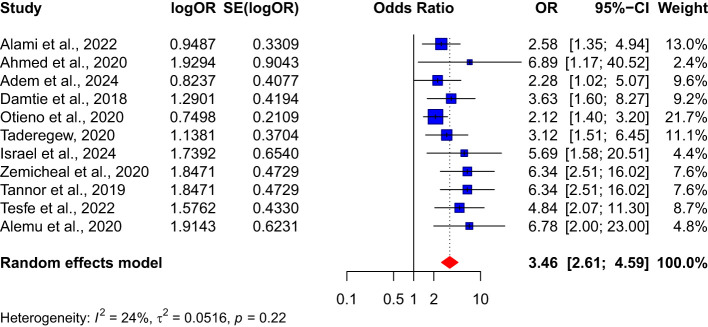
Association between hypertension and nephropathy among diabetes patients.

The results of the meta-analysis showed a significant association between hypertension and nephropathy among diabetes patients. The pooled effect size from 11 studies indicated that diabetes patients with hypertension are more than three times at risk of developing nephropathy compared to those without hypertension OR:3.46 (95% CI: 2.61-4.59). Furthermore, the analysis identified no substantial heterogeneity, evidenced by an I^2^ value of 24% (see **Figure 9**).

### 3.8 Association between duration of diabetes and nephropathy

**Figure 10:**
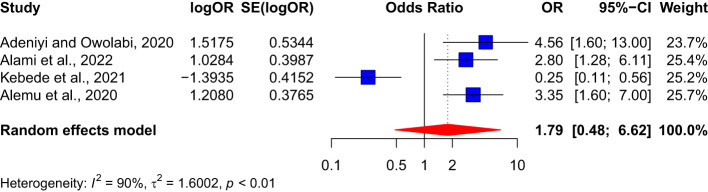
Association between duration of diabetes and nephropathy.

The pooled effect size from four studies indicated that having diabetes for more than 10 years does not have a significant association with developing nephropathy, with OR: 1.79 (95% CI: 0.48–6.62) (See **Figure 10**). However, the results had substantial heterogeneity, as indicated by an I² value of 90%.

### 3.9 Publication bias and sensitivity analysis

**Figure 11:**
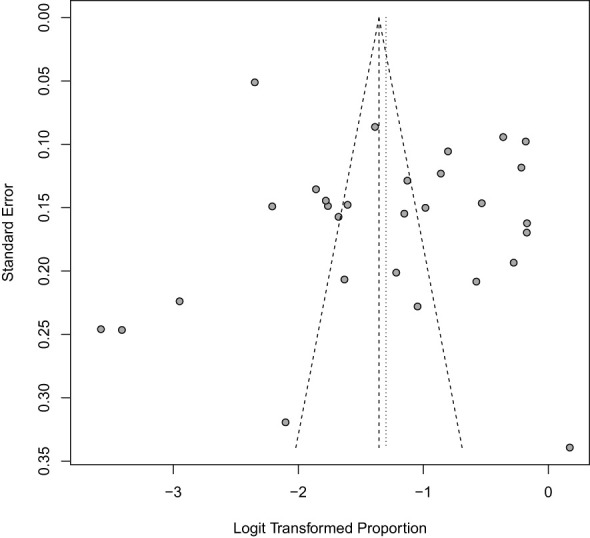
Funnel plot for publication bias in the included studies.

**Figure 12:**
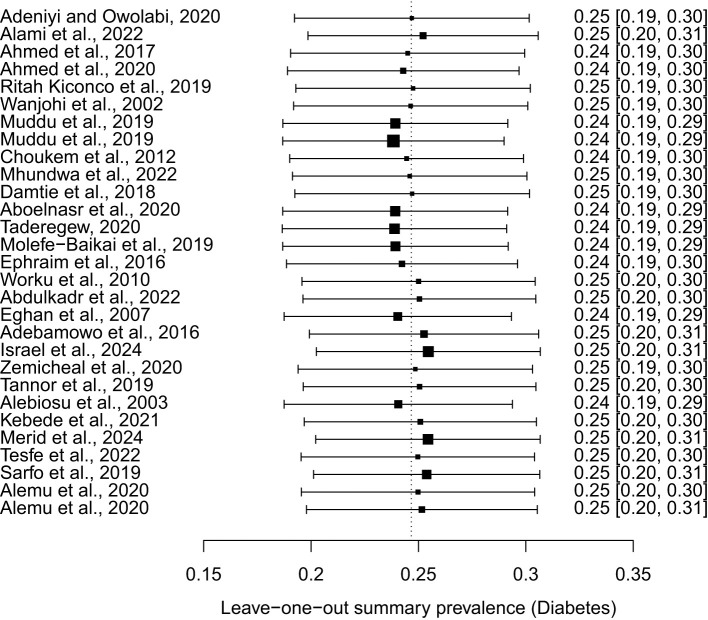
Leave−one−out summary prevalence (Diabetes).

The funnel plot indicated significant asymmetry (**Figure 11**). However, a leave-one-out analysis of the prevalence of nephropathy among diabetes patients was conducted to assess the impact of individual studies on the pooled prevalence. The results showed that the weighted prevalence remained consistent with the original summary prevalence. This suggests that the conclusion drawn from this meta-analysis should be interpreted with caution (See **Figure 12**).

### 3.10 Risk of bias assessment for the included studies

The thirty-four (34) included articles were assessed for risk of bias and methodological quality by adapting the JBI critical appraisal checklist for studies reporting prevalence data. Each checklist was assigned a score of 1, and the overall score was 9. The articles were appraised, and each study obtained a maximum score of 9. On the quality scale, a score between ≤5 was deemed ‘high risk’’, six was deemed ‘moderate risk’’ and a score of ≥7 was deemed ‘low risk’’. Only 4(11.8%) of the articles were deemed to have a ‘high risk’’ of bias, whereas 26(76.5%) of the articles fell into the ‘low risk’’ bias. Finally, 4(11.8%) of the articles were determined to have a moderate risk of bias with a score of 6 out of 9 (**Supplementary Table 4**).

## 4 Discussion

The prevalence of nephropathy among diabetes mellitus patients in Africa, as well as the risk factors associated with its development, are underreported. Diabetic nephropathy is one of the most prevalent and dangerous diabetes mellitus complications. However, there has been a paucity of reliable statistics on its prevalence among diabetes mellitus patients and the risk factors contributing to its development in African regions. This paucity of data could be associated with a lack of serious documentation or renal registries (56). The main objectives of this study were to evaluate the prevalence of nephropathy among diabetes mellitus patients in Africa and further understand the contributing factors to the development of nephropathy. The current study identified 34 relevant articles published from 2000 to 2024 on the prevalence of nephropathy among diabetes mellitus patients in Africa and various risk factors enhancing its development.

The pooled prevalence of diabetes nephropathy reported was from 28 eligible studies, and as clearly observed in the result, the pooled prevalence of diabetes nephropathy was 21% (95% CI 16-28), indicating a substantial burden of diabetes nephropathy in Africa. This prevalence is higher than reported in the systematic review and meta-analysis conducted (57) in Asia. The significant heterogenicity (I² = 98%) observed in the study highlights the variation in study populations and geographical locations.

Furthermore, the prevalence of nephropathy varied between the two types of diabetes mellitus studied. In type 1, the pooled prevalence was significantly higher, 46% (18-77, I² = 98%), than the 20% (95% CI: 14-27, I² = 98%) prevalence observed among type 2 diabetes patients. A report by Wu et al. (58) stated that adults with Type 2 diabetes mellitus had a prevalence of 38.3% from 2007-2012, whereas Elhafeez et al. (59) reported that the pooled prevalence among type 2 diabetes mellitus patients was 24.7% (95% CI 23.6–25.7%). Contrary to these reports, our findings from the review and meta-analysis of 28 articles published between 2002 and 2024 showed a higher prevalence of diabetes nephropathy among type 1 diabetes patients. This disparity may be attributed to differences in disease duration, glycaemic control, and genetic predisposition. The higher prevalence in type 1 diabetes patients underscores the need for intensified monitoring and early intervention. To further elaborate on the factors that might contribute to heterogeneity observed in the results, it is important to emphasize variation in the geographical location and countries of the 28 studies represented in the analysis. Additionally, the characteristics of the study population, including variations in age, sex, ethnicity, genetic variably and socioeconomic status, contributed to the heterogeneity (60–62). The study design also varied, with both cross-sectional and cohort studies included. Furthermore, differences in methodology, such as data collection tools, added to the heterogeneity. The duration and severity of the disease also impacted on the prevalence of diabetic nephropathy (61), contributing to the variations observed. Lastly, differences in prevalence between type 1 and type 2 diabetes patients also played a significant role in heterogeneity. To address possible bias, a funnel plot which may indicate that studies with significant results are overrepresented in literature, while those with non-significant results are underrepresented was conducted. The funnel plot revealed significant asymmetry, suggesting potential publication bias. To address this concern, a leave-one-out sensitivity analysis was performed, which removed each study individually from the meta-analysis and recalculated the pooled prevalence. The results showed that the weighted prevalence remained consistent with the original summary prevalence, indicating that no single study had a disproportionate influence on the overall estimate. While this finding provides reassurance about the robustness of the results, caution is still warranted when interpreting the findings.

A 5-year retrospective study by Zhang et al. (63) stated that the prevalence of nephropathy and changes in renal activity was greater in women than men, indicating gender differences in the prevalence of nephropathy in Type 2 diabetes. Moreover, it was noted that women showed a more pronounced loss in renal function with an increase in follow-up duration. In men, the prevalence of nephropathy in type 2 diabetes mellitus patients was substantially linked with age, insulin resistance, and hypertension. Age and the length of diabetes were associated in female patients.

Our study’s findings show no significant differences in the pooled prevalence of nephropathy between male and female diabetes mellitus patients. According to the meta-analysis, the pooled prevalence of diabetes among female patients is 35% (95% CI: 26-45, I² = 97%), whereas the pooled prevalence among male patients is 36% (95% CI: 28-46, I² = 96%).

Equally observed in this study was a differential variation in the geographical distribution of diabetes nephropathy prevalence among diabetes mellitus patients in Africa. The main observation was the highest prevalence in North Africa, followed by South Africa, while the lowest was in West Africa despite having the highest recorded sample size. The disparity in the regions can be attributed to genetic factors (64) and systemic factors like Western cultural influences, population changes, low-quality healthcare, and personal factors such as poverty, educational status, perceptions about the disease, diet, and lifestyle, such as exercise (65). The Western cultural influences on North Africa are on a high scale compared to those in West Africa because of their geographical proximity, which contrasts what is obtainable in the West African region. Studies have also reported difficulties in educating lifestyle intervention programs due to existing patients’ habits, including cultural diet and eating patterns, being more evident in West Africa than in North Africa (60). Population changes have to do with the migration of people from rural to urban cities, where they consume pre-packed foods and are more susceptible to the disease. Most West Africans are rural dwellers, where consumption of pre-packed foods is not typical and where a sedentary lifestyle is absent compared to other regions of Africa (65).

Age, gender, duration of diabetes, hypertension, and poor glycaemic control are among the various factors reported to contribute to the severity of diabetes mellitus by orchestrating the development of diabetes commodities.

In this study, we highlighted hypertension as a critical risk factor for nephropathy among diabetes patients. The meta-analysis revealed a significant association between hypertension and nephropathy among diabetes patients. This finding suggests that hypertension significantly increases the risk of developing nephropathy by more than threefold. The low heterogeneity (I² = 24%) among the 11 studies included in this analysis lends credibility to this Association. The strong link between hypertension and nephropathy underscores the importance of blood pressure management among diabetes mellitus patients. Contrary to common observation, our analysis did not show any significant association between the duration of diabetes and the risk of developing nephropathy (OR: 1.79, 95% CI: 0.48-6.62). However, substantial heterogeneity (I² = 90%) among the four studies included in this analysis might be attributed to variations in the study populations, thus suggesting that this finding should be interpreted cautiously.

### 4.1 Strengths

This systematic review includes an extensive search strategy covering multiple database sources, which reduces publication bias and ensures relevant studies are captured. Reproducible methodology, including predefined inclusion and exclusion criteria, enhances the reliability of the study. Also, the rigorous quality assessment using established tools like PRISMA risk analysis scoring to evaluate the quality of each included study, thereby increasing the credibility of the conclusion. Conducting a meta-analysis to provide a quantitative summary of the findings across studies. Identifying and analyzing sources of heterogeneity was also strength in this study.

### 4.2 Limitations

Not all countries in Africa had representative articles in the included studies. Many studies were excluded based on the exclusion criteria. Risk factors within each country responsible for their prevalence were not considered.

### 4.3 Conclusion and recommendation

The current study looked at the prevalence of nephropathy among diabetic mellitus (DM) patients and the risk variables in different parts of Africa. West Africa had the lowest prevalence of nephropathy among people with diabetes, whereas North Africa had the highest frequency of the condition. Consequently, investigations on nutritional determinants, patient adherence to diet adjustments, Western cultural impacts on African dietary consumption, and knowledge of nephropathy management among diabetes mellitus patients should be prioritized in Africa to lessen the burden of the disease. The possibility of West African dietary habits as a contributory factor to the lower burden of nephropathy in West Africa is worthy of investigation and will serve as a guideline for other continents with higher incidences of diabetes mellitus nephropathy.

## Data Availability

The original contributions presented in the study are included in the article/**Supplementary Material**. Further inquiries can be directed to the corresponding author.
