# Supplementary material for: Prevalence of nephropathy among patients with diabetes mellitus in Africa: a systematic review and meta-analysis
Source: Front Clin Diabetes Healthc. 2025 Apr 25;6:1551088. doi: 10.3389/fcdhc.2025.1551088 (PMC12061956; doi:10.3389/fcdhc.2025.1551088)
Supplement: Supplementary file 1 [file Table1.docx]

**Supplementary file 1: Search strategy for the study**

| Search Terms | Synonyms | Boorlean operators | Search database |
| --- | --- | --- | --- |
| Prevalence | 1. Frequency 2. Occurrence | OR | Pubmed |
| Nephropathy | 1. Kidney disease 2. Chronic kidney disease 3. Renal Insufficiency 4. Proteinuria 5. Kidney failure 6. Macroalbuminuria 7. Macro-albuminuria 8. Micro-albuminuria 9. Microalbuminuria 10. renal impairment 11. End stage renal insufficiency 12. End stage renal failure 13. End stage kidney failure 14. Renal failure 15. kidney injury 16. Nephrosis 17. Glomerulonephritis 18. Diabetes kidney diseases 19. end stage kidney diseases | OR | " |
| Diabetes mellitus | 1. Type 1 diabetes mellitus 2. Type 2 diabetes mellitus 3. Diabetes mellitus 4. T2DM 5. T1DM 6. hyperglycaemia 7. Type 1 diabetes 8. Type 2 diabetes 9. Juvenile diabetes 10. Insulin dependent diabetes 11. Non insulin dependent diabetes | OR | " |
| Contributing factors | 1. determinants 2. risk factor 3. association 4. exacerbating | OR | " |
| Africa | 1. Algeria 2. Angola 3. Benin 4. Botswana 5. Burkina Faso 6. Burundi 7. Cameroon 8. Canary Islands 9. Cape Verde 10. Cabo Verde 11. Central African Republic 12. Chad 13. Comoros 14. Congo 15. Democratic Republic of the Congo 16. Republic of the Congo 17. Djibouti 18. Egypt 19. Equatorial Guinea 20. Eritrea 21. Eswatini 22. Swaziland 23. Ethiopia 24. Gabon 25. Gambia 26. Ghana 27. Guinea 28. Guinea-Bissau 29. Ivory Coast 30. Côte d'Ivoire 31. Kenya 32. Lesotho 33. Liberia 34. Libya 35. Madagascar 36. Malawi 37. Mali 38. Mauritania 39. Mauritius 40. Morocco 41. Mozambique 42. Namibia 43. Niger 44. Nigeria 45. Rwanda 46. São Tomé and Príncipe 47. Senegal 48. Seychelles 49. Sierra Leone 50. Somalia 51. South Africa 52. South Sudan 53. Sudan 54. Tanzania 55. Togo 56. Tunisia 57. Uganda 58. Zambia 59. Zimbabwe 60. west-africa 61. west africa 62. sub-saharan africa 63. sub saharan africa 64. sub saharan african 65. sub-saharan african 66. west african 67. east africa | OR |  |
| Search Period | January 1, 2000 to August 2024 |  |  |

**Supplementary file 2: Summary and List of excluded articles**

| **S/N** | **Author** | **Title** | **Decision** | **Reason** |
| --- | --- | --- | --- | --- |
|  | Bentata et al., 2015 | Diabetic kidney disease and vascular comorbidities in patients with type 2 diabetes mellitus in a developing country | Excluded | Population of DM patients already diagnosed with kidney diseases were included |
|  | Blaslov et al., 2014 | Waist-to-height ratio is independently associated with chronic kidney disease in overweight type 2 diabetic patients | Excluded | The study included population with established kidney diseases. The study is equally an association study with anthropometric parameters |
|  | Mpondo et al., 2016 | Prevalence of chronic kidney disease in diabetic adult out-patients in Tanzania | Excluded | The study is a correspondence article |
|  | Al-Ozairi et al., 2021 | Prevalence of Cardiovascular and Renal Co morbidities in Patients with Type 2 Diabetes in the Gulf, a Cross-sectional Observational Study | Excluded | Wrong outcome |
|  | Ramaphane et al., 2021 | Prevalence and Factors Associated with Microalbuminuria in Pediatric Patients with Type 1 Diabetes Mellitus at a Large Tertiary-Level Hospital in Botswana | Excluded | Secondary study and a retrospective cross-sectional study |
|  | Bentata et al., 2016 | Albuminuria in type 2 diabetes mellitus: from remission to progression | Excluded | Intervention study: patients already attending nephrology clinic, and the remission of macro and microalbuminuria were the outcome |
|  | Keeton et al., 204 | Renal outcome of type 2 diabetes in South Africa—a 12-year follow-up study | Excluded | population<100 |
|  | El-Shal et al., 2014 | Adiponectin gene polymorphisms in Egyptian type 2 diabetes mellitus patients with and without diabetic nephropathy | Excluded | Study assessed adiponectin gene polymorphism among T2DM patients and its relation to serum adiponectin level |
|  | Alıcı1 and Genç, 2022 | The pattern of dyslipidemia among Somali type 2 diabetic patients: a cross-sectional study | Excluded | Wrong outcome |
|  | Alebiosu, 2003 | Clinical diabetic nephropathy in a tropical African population | Excluded | Only established cases of DN among DM patients were considered which will not give a view of DN prevalence among DM patients |
|  | Motshwari et al., 20021 | Novel Whole Blood MicroRNAs Predicting Chronic Kidney Disease in South Africans with Hypertension and Diabetes Mellitus | Excluded | study is on the association of miRNAs to CKD irrespective of the conventional risk factors |
|  | George et al., 2023 | Leveraging the South African Diabetes Prevention Programme to screen for chronic kidney disease: an observational study | Excluded | study not specific on the prevalence of DN among DM patients |
|  | Assaad-Khalil et al., 2024 | Neutrophil-to-Lymphocyte Ratio as a Marker for Cardiac Autonomic Neuropathy in Egyptian Patients with Type 2 Diabetes: A Cross-Sectional Study | Excluded | Participants were less than 100 (only 90 patients in the study) |
|  | Siwy et al., 2014 | Multicentre prospective validation of a urinary peptidome-based classifier for the diagnosis of type 2 diabetic nephropathy | Excluded | Wrong outcome |
|  | Ekrikpo, et al., 2017 | Clinical Utility of Urinary 𝛽2-Microglobulin in Detection of Early Nephropathy in African Diabetes Mellitus Patients | Excluded | Methodological study on early detection of diabetes nephropathy |
|  | Ploth et al., 2018 | Prevalence of CKD, Diabetes, and Hypertension in Rural Tanzani | Excluded | The study assessed prevalence of chronic kidney disease (CKD), diabetes mellitus (DM) and hypertension independently and could not associate CKD to DM |
|  | Yarhere et al., 2020 | Microalbuminuria in type 1 diabetes mellitus children in University of Port Harcourt Teaching Hospital, Nigeria | Excluded | Small population of children (just 22 participant) |
|  | Utura and Fikrie, 2022 | Prevalence and associated factors of diabetes mellitus among Governmental Civil Servants at Guji Zone, Oromia Region, Ethiopia, 2021. A community-based cross-sectional study | Excluded | Study focused on the prevalence of DM |

**Supplementary file 4: Quality Assessment of Studies**

| **S/N** | **First Author’s Surname** | **Year** | **Tittle** | **Country** | **Geographical region** | **Study settings** | **Sample size** | **Mean age** | **Type of study** | **Quality Assessment Score** |
| --- | --- | --- | --- | --- | --- | --- | --- | --- | --- | --- |
|  | Adeniyi and Owolabi^22^ | 2020 | Cross-sectional study of diabetes kidney disease in the Eastern Cape, South Africa | South Africa | South Africa | Facility based | 327 | ≥30 | Cross- sectional study | Low risk |
|  | Alami et al^23^ | 2022 | Prevalence and risk factors of chronic complications among patients with type 2 diabetes mellitus in Morocco: a cross-sectional study | Morocco | North Africa | Facility based | 505 | 57.27±10.74 | Cross-sectional study | Low risk |
|  | Ahmed et al^47^ | 2017 | The relationship between diabetic retinopathy and nephropathy in Sudanese adult with diabetes: population based study | Sudan | Northeast Africa | Facility based | 316 | 58 ±10 | Descriptive Cross-sectional | Moderate risk |
|  | Ahmed et al^48^ | 2020 | Early Diabetic Nephropathy and Retinopathy in Patients with Type 1 Diabetes Mellitus Attending Sudan Childhood Diabetes Centre | Sudan | North East Africa | Facility based | 100 | >10 | Cross sectional study | Low risk |
|  | Ritah Kiconco et al^24^ | 2019 | Microalbuminuria and Traditional Serum Biomarkers of Nephropathy among Diabetic Patients at Mbarara Regional Referral Hospital in South Western Uganda | Southwestern Uganda | East Africa | Facility based | 140 | 45-54 | Cross sectional study | High risk |
|  | Adem et al^49^ | 2024 | Prevalence of chronic kidney disease and its associated factors among diabetes mellitus patients in Dessie Referral Hospital, South Wollo, Ethiopia | Ethiopia | East Africa | Facility based | 267 | ≥ 18 | Cross-sectional study | Low risk |
|  | Wanjohi et al^50^ | 2002 | Nephropathy in patients recently diagnosed with type 2 diabetes melitus in black Africans | Kenya | East African | Facility based | 100 | 53.7±9.3 | Cross sectional study | High risk |
|  | Muddu et al^25^ | 2019 | Utility of albumin to creatinine ratio in screening for microalbuminuria among newly diagnosed diabetic patients in Uganda: a cross sectional study | Uganda | East African | Facility based | 175 | 46±15 | Cross sectional study | Low risk |
|  | Choukem et al^51^ | 2012 | Comparison of different blood pressure indices for the prediction of prevalent diabetic nephropathy in a sub-Saharan African population with type 2 diabetes | Cameroon | Central Africa | Facility based | 420 |  | Cross-sectional study | High risk |
|  | Bentata et al^36^ | 2015 | Diabetic Kidney Disease and Vascular Comorbidities in Patients with Type 2 Diabetes Mellitus in a Developing Country | Morocco | North Africa | Facility based | 637 | 58.5 ± 10.8 | Prospective Cohort study | Moderate risk |
|  | Mhundwa et al^52^ | 2023 | The prevalence of chronic kidney disease among type 2 diabetes mellitus patients in central South Africa | South Africa | South Africa | Facility based | 224 | 62.5 | Cross-sectional study | Low risk |
|  | Damtie et al^53^ | 2018 | Chronic Kidney Disease and Associated Risk Factors Assessment among Diabetes Mellitus Patients at A Tertiary Hospital, Northwest Ethiopia | Ethiopia | East Africa | Facility based | 229 | 47±15.7 | Cross-sectional study | Low risk |
|  | Aboelnasr et al^54^ | 2020 | Diabetic Kidney Disease in Patients Newly Diagnosed withType-2 Diabetes Mellitus: Incidence and Associations | Egypt | North Africa | Facility based | 153 | 49.1 | Cross-sectional study | Low risk |
|  | Otieno et al^26^ | 2020 | The burden of unrecognised chronic kidney disease in patients with type 2 diabetes at a county hospital clinic in Kenya: implications to care and need for screening | Kenya | East Africa | Facility based | 385 | 63.3 | Cross-sectional study | Low risk |
|  | Taderegew^55^ | 2020 | Assessment of renal impairment using estimated glomerular filtration rate among type 2 diabetes mellitus patients in North-East Ethiopia: a cross-sectional study | Ethiopia | East Africa | Facility based | 422 |  | Cross-sectional study | Low risk |
|  | Molefe-Baikai et al^27^ | 2019 | The Prevalence of Microalbuminuria and Associated Factors among Patients with Type 2 Diabetes Mellitus in Botswana | Botswana | East Africa | Facility based | 289 | 42–53 | Cross-sectional study | High risk |
|  | Ephraim et al^28^ | 2016 | Chronic Kidney Disease Stages among Diabetes Patients in the Cape Coast Metropolis | Ghana | West Africa | Facility based | 200 |  | Cross-sectional study | Moderate risk |
|  | Worku et al^56^ | 2010 | Patterns of diabetic complications at jimma university specialized hospital, southwest ethiopia | Ethiopia | East Africa | Facility based | 305 |  | Cross-sectional study | Moderate risk |
|  | Abdulkadr et al^57^ | 2022 | Chronic Kidney Disease and Associated Factors among Diabetic Patients at the Diabetic Clinic in a Police Hospital, Addis Ababa | Ethiopia | East Africa | Facility based | 362 | 55.4±13.63 | Cross-sectional study | Low risk |
|  | Eghan et al^29^ | 2007 | Prevalence and predictors of microalbuminuria in patients with diabetes mellitus:across-sectional observational study in kumasi, ghana | Ghana | West Africa | Facility based | 109 | 54.1±10.9 | Cross-sectional study | Low risk |
|  | Adebamowo et al^38^ | 2016 | Impact of Type 2 Diabetes on Impaired Kidney Function in Sub-Saharan African Populations | Ghana, Kenya and Nigeria | West and East Africa | Facility and Community based | 4815 | 48 | Case-control | Low risk |
|  | Israel et al^50^ | 2024 | Out of sight: chronic kidney diseases among diabetic patients attending care and follow up. Findings from pastoralist health facilities of Southern Ethiopia | Ethiopia | East Africa | Facility based | 626 |  | Cross-sectional study | Low risk |
|  | Zemicheal et al^39^ | 2020 | Determinants of Diabetic Nephropathy among Diabetic Patients in General Public Hospitals of Tigray, Ethiopia, 2018/19 | Ethiopia | East Africa | Facility based | 840 |  | Case-control | Low risk |
|  | Tannor et al^59^ | 2019 | Prevalence and predictors of chronic kidney disease among Ghanaian patients with hypertension and diabetes mellitus: A multicenter cross‐sectional study | Ghana | West Africa | Facility based | 388 |  | Cross-sectional study | Low risk |
|  | Alebiosu et al^16^ | 2003 | Morbidity in relation to stage of diabetic nephropathy in type-2 diabetic patients | Nigeria | West Africa | facility based | 465 |  | Retrospective study | Low risk |
|  | Kebede et al^34^ | 2021 | Incidence of Diabetic Nephropathy and Its Predictors among Type 2 Diabetes Mellitus Patients at University of Gondar Comprehensive Specialized Hospital, Northwest Ethiopia | Ethiopia | East Africa | facility based | 467 |  | Retrospective follow-up study | Low risk |
|  | Merid et al^61^ | 2024 | Incidence and Predictors of Diabetic Nephropathy among Type 2 Diabetes Mellitus Patients, Southern Ethiopia | Ethiopia | East Africa | Facility based | 532 |  | Retrospective follow up study | Low risk |
|  | Tesfe et al^30^ | 2022 | The proportion of chronic kidney disease and its associated factors among adult diabetic patients at Tibebe Ghion Specialized Hospital, Bahir Dar, Ethiopia | Ethiopia | East Africa | Facility based | 329 |  | Cross sectional study | Low risk |
|  | Sarfo et al^37^ | 2019 | Estimated glomerular filtration rate predicts incident stroke among Ghanaians with diabetes and hypertension | Ghana | West Africa | Facility based | 422 | 49.7±12.2 | Prospective cohort study | Low risk |
|  | Alemu et al^31^ | 2020 | Prevalence of Chronic Kidney Disease and Associated Factors among Patients with Diabetes in Northwest Ethiopia: A Hospital-Based Cross-Sectional Study | Ethiopia | East Africa | Facility based | 272 | 51.67 ±13.75 | Cross sectional study | Low risk |
|  | Ahmed et al^35^ | 2022 | Incidence and predictors of chronic kidney disease in type-II diabetes mellitus patients attending at the Amhara region referral hospitals, Ethiopia: A follow-up study | Ethiopia | East Africa | Facility based | 415 | 56.13±10.2 | Retrospective follow-up study | Low risk |
|  | Tamru et al^62^ | 2020 | Incidence of diabetic nephropathy in patients with type 2 diabetes mellitus at a tertiary healthcare setting in Ethiopia | Ethiopia | East Africa | Facility based | 346 | 56.70±10.48 | Retrospective follow-up study | Low risk |
|  | Machingura et al^60^ | 2017 | Prevalence of and Factors Associated with Nephropathy in Diabetic Patients Attending an Outpatient Clinic in Harare, Zimbabwe | Zimbabwe | Southern Africa | Facility based | 344 | 57.6±14.8 | Cross sectional study | Low risk |
|  | Hamat et al^32^ | 2016 | Profile of diabetic nephropathy at the National General Reference Hospital of N'Djamena (Chad) | Chad | Central Africa | Facility based | 181 | 58.7 | Cross-sectional study | Low risk |
